# Evaluation of the rubella surveillance system in South Africa, 2016–2018: A cross-sectional study

**DOI:** 10.1371/journal.pone.0287170

**Published:** 2023-06-23

**Authors:** Fhatuwani Gavhi, Alex De Voux, Lazarus Kuonza, Nkengafac Villyen Motaze

**Affiliations:** 1 National Institute for Communicable Diseases, A Division of the National Health Laboratory Service, Johannesburg, Gauteng, South Africa; 2 School of Health Systems and Public Health, University of Pretoria, Pretoria, Gauteng, South Africa; 3 Division of Epidemiology and Biostatistics, School of Public Health, University of Cape Town, Cape Town, Western Cape, South Africa; 4 School of Public Health, University of Witwatersrand, Johannesburg, Gauteng, South Africa; 5 Medicine Usage in South Africa, School of Pharmacy, Faculty of Health Sciences, North-West University, Potchefstroom, North-West, South Africa; National Health Laboratory Service, SOUTH AFRICA

## Abstract

**Background:**

Rubella is a leading vaccine-preventable cause of birth defects. We conducted this study to evaluate the rubella surveillance system in South Africa from 2016 to 2018. The rubella surveillance system had not been evaluated since its inception; therefore, a formal evaluation is necessary to assess key attributes and to ascertain the extent to which the system achieves its objectives.

**Methods:**

We conducted a cross-sectional study to assess the usefulness, simplicity, positive predictive value, timeliness, and data quality of the rubella surveillance system from 2016 to 2018. We reviewed retrospective rubella surveillance data and conducted a survey with key stakeholders of the system. We compiled a summary report from the survey and calculated the annualized detection rate of rubella and non-rubella febrile rash, positive predictive value, the proportion of complete records, and timeliness between the surveillance steps. We compared our results with recommended performance indicators from the 2015 revised World Health Organization African regional guidelines for measles and rubella surveillance.

**Results:**

The rubella surveillance system was useful but weak in terms of simplicity. The annualized detection rate of rubella febrile rash was 1.5 per 100,000 populations in 2016, 4.4 in 2017, and 2.1 in 2018. The positive predictive value was 29.1% in 2016, 40.9% in 2017, and 32.9% in 2018. The system did not meet the timeliness goal in the health facility component but met this goal in the laboratory component. The system had poor data quality, particularly in the health facility component.

**Conclusions:**

The rubella surveillance system was useful, although it was not simple to use and had low PPV, poor timeliness, and poor data quality. Efforts should be made to improve the system’s simplicity, PPV, timeliness, and data quality at the facility level.

## Introduction

Rubella is a contagious infection caused by a virus of the genus *Rubivirus* in the *Togaviridae* family. Rubella is transmitted through airborne droplets when infected individuals cough or sneeze [1]. Although rubella causes mild symptoms like fever and rash in children and adults, infection during pregnancy can result in serious complications such as miscarriage, fetal death, and congenital abnormalities known as Congenital Rubella Syndrome (CRS) [1, 2]. CRS birth abnormalities may include eye and heart defects, hearing impairment, microcephaly or late-onset manifestations like autism, diabetes mellitus, and thyroiditis [3, 4]. Women who contract rubella during the first trimester of pregnancy have up to a 90% chance of giving birth to infants with CRS [1]. Although rubella does not have a specific treatment, it can be prevented through vaccination [5]. Due to the global burden of CRS and the proven efficacy and safety of rubella vaccination, the World Health Organization (WHO) recommends that countries introduce rubella vaccinations in their immunization programs [5]. Over the years, CRS incidence and rubella infection have been decreasing in countries that have rubella-containing vaccines in their immunization programs [5, 6]. The WHO African region has the highest annual incidence of CRS, however, only 14 African countries had introduced rubella vaccines by 2017 [7, 8]. South Africa currently does not have rubella-containing vaccines in the Expanded Programme on Immunization (EPI); nonetheless, rubella vaccines are administered in the private health sector [9].

It is estimated that more than 100 000 babies are born with CRS globally each year [7]. Africa is one of the regions with a high burden of rubella infection and CRS [5]. In 2015, 45.1% and 47% rubella seroprevalence was reported among children and women of childbearing age in Africa [10]. In South Africa, rubella is endemic and 43% seroprevalence was reported between 2016 and 2018 [11]. The exact burden of CRS is unknown; however, it is estimated that approximately 660 cases occur every year [12–14]. Through the sentinel surveillance system, a total of 95 laboratory-confirmed CRS cases were reported between January 2010 and December 2017 in South Africa [15].

To accelerate rubella elimination and control goals, WHO recommends that countries conduct rubella surveillance to keep track of cases [2]. Surveillance is a critical element in disease prevention and control, as it provides essential epidemiological data to facilitate public health action [16, 17]. Rubella surveillance is necessary to estimate incidence, prevalence, trends over time, detect outbreaks, identify at-risk populations, and inform decision-makers on prevention and control strategies [18, 19]. Measles elimination strategies are currently used by countries and international partners to achieve the rubella elimination and control goals [2, 5, 18, 19]. These strategies include introduction of the rubella vaccine in routine vaccination schedules and the integration of measles, rubella, and CRS surveillance systems [2, 19, 20]. In South Arica, it is not known when rubella surveillance was started, however, the earliest available surveillance data is from 1998 [12–14]. Rubella surveillance was discontinued from 2013 to 2014 and re-established in May 2015. Periodic evaluation of any surveillance system is essential to assess effectiveness and to ascertain if the system is achieving its stipulated objectives. The rubella surveillance system in South Africa has not been evaluated since its inception, and South Africa is planning to introduce the rubella vaccine in the EPI. Therefore, it is necessary to assess the functionality of the rubella surveillance system. The purpose of this study was to assess key attributes of the rubella surveillance system in South Africa and to ascertain the extent to which the system achieves stated objectives and make recommendations for improvement.

## Materials and methods

### Study design and setting

We conducted a cross-sectional study from 2016 to 2018 that entailed retrospective rubella data analysis and a survey that collected data from stakeholders in South Africa. South Africa has nine provinces and 52 districts and a total population size of 59.6 million was recorded in 2018 [21]. The public health system of South Africa is structured into three levels with national, provincial, and district Departments of Health (DoH). There are four tiers of health facilities: tertiary, regional, and district hospitals and primary healthcare facilities. We conducted a survey of selected users of the rubella surveillance system from health facilities in Gauteng and Limpopo provinces. Gauteng province had a population of 15.5 million individuals which represented the largest share (26.0%) of the South African population in 2018 [21]. WHO South Africa country office, National Institute for Communicable Diseases (NICD), National Department of Health (NDoH) are also located in the Gauteng province. Limpopo province had a population size of 5.9 million [21] and was the fifth most populated province in South Africa following Eastern Cape, Western Cape, KwaZulu-Natal and Gauteng provinces. Gauteng and Limpopo provinces share a border, and they are both divided into five districts.

### Rubella surveillance system in South Africa

Rubella surveillance is entirely laboratory-based and is coordinated by the Center for Vaccines and Immunology (CVI), NICD, a division of the National Health Laboratory Services (NHLS). The NHLS is the largest diagnostic pathology service provider in South Africa, and provides laboratory services to more than 80% of the population, through a network of over 260 laboratories distributed across all provinces of the country [22]. All laboratory tests done in NHLS laboratories are captured electronically in the Corporate Data Warehouse (CDW), a central data repository of the NHLS.

Rubella symptoms are like those that are caused by measles virus. Therefore, the practice is that blood samples are collected from suspected measles cases within all four tiers of health facilities and sent to CVI, NICD for measles and rubella serological testing. Samples are collected in health facilities using suspected measles case definition adopted from WHO. A suspected measles case is any person with fever (≥38°C) and maculopapular (non-vesicular) generalized rash and any of the three C’s: cough, coryza or conjunctivitis or anyone whom a clinician suspect measles infection [23]. A confirmed rubella case definition adopted from WHO is used to classify cases after laboratory testing. A laboratory-confirmed rubella case is a suspected case with a positive blood test for rubella-specific IgM [23]. After laboratory testing, NICD shares the results with the clinicians within all four tiers of health facilities, district, provincial and national DoH and WHO.

For each suspected case, healthcare workers complete surveillance tools (case investigation form and notifiable medical condition form) with epidemiological data [24]. Completed surveillance tools are sent to the district, provincial, and national DoH, as well as to the NICD via email or the notifiable medical conditions electronic platform [24].

### Rubella surveillance system evaluation framework

We evaluated this surveillance system using the Updated Guidelines for Evaluating a Public Health Surveillance System published by the Centers for Disease Control and Prevention (CDC), Atlanta, United States of America [25]. The CDC guidelines describe attributes that a surveillance system should have to be effective and efficient. To determine the quality and effectiveness of the system, we compared results of our evaluation with the recommended performance indicators from the 2015 revised WHO African regional guidelines for measles and rubella surveillance [23].

### Operational definitions

**Table:** 

**Definition**	**Description**
Facility level	refers to health institutions that include community health centres and hospitals
District level	refers to district Department of Health that were included in this study (Capricorn, Waterberg, City of Ekurhuleni, and City of Tshwane)
Provincial level	refers to Gauteng and Limpopo provincial Department of Health
National level	refers to the National Department of Health and National Institute for Communicable Diseases
Facility turnaround time	refers to the time taken between samples collection in the health facilities and samples arrival at National Institute for Communicable Diseases laboratory
Laboratory turnaround time	refers to the time taken between receipt of the samples in the laboratory and issuing of the rubella test results
Complete record	refers to any record that had cases’ date of birth, sex, facility name, facility’s ward name, date of symptoms onset, date of samples collection, district name, province name, epidemiology number, test week, test month, test year, sample type, referring laboratory name, test laboratory number, laboratory reference number, test registration date, and date of test results review
Clinical characteristics	refers to variables such as cases’ date of birth, sex, name of the facility, facility’s ward name, date of symptoms onset, date of samples collection, name of the district, name of the province, epidemiology number and presence of case investigation form on the national rubella surveillance database
Laboratory characteristics	refers to variables such as test week, test month, test year, sample type, name of referring laboratory, test laboratory number, laboratory reference number, test registration date and date of test results review on the national rubella database

### Study population and sampling

For the survey, we included healthcare workers involved in coordinating measles and rubella surveillance systems at WHO South Africa, NICD, national, provincial and district DoH and those involved in diagnosing, recording, and notifying health authorities of suspected measles cases in public health facilities of Gauteng and Limpopo provinces. We conveniently selected Gauteng and Limpopo provinces because they were easily accessible to the study team. We purposively selected districts and health facilities that were included in the 2017 WHO comprehensive EPI, data quality, essential vaccines management and in-depth surveillance review. We conducted survey in seven health facilities, including hospitals and community health centres, from Gauteng and Limpopo provinces that were included in 2017 WHO review. We interviewed measles and rubella laboratory manager, data administrator, and personnel who test samples at CVI and EPI managers at the selected districts. We also targeted to interview measles and rubella surveillance coordinators at WHO South Africa country office, NDoH, and Gauteng and Limpopo EPI provincial managers. At the facility-level, we purposively included all medical doctors and nurses at outpatient department and paediatric ward.

### Measurements

We measured key attributes of the surveillance system through survey and analysis of the rubella surveillance database (Table 1).

**Table 1:** Measurements of attributes of the rubella surveillance system.

Attributes	Measurement	Source of data	
Quantitative		Rubella surveillance database	Cross-sectional survey
Positive predictive value	Calculation of the proportion of cases that tested rubella IgM positive from the total number of reported suspected cases that were tested for rubella infection.	Yes	
Timeliness	Calculation of facility and laboratory turnaround times (in days).	Yes	
Data quality	Calculation of the proportion of complete records on the rubella surveillance database. Complete record was defined as any record that had cases’ date of birth, sex, facility name, facility’s ward name, date of symptoms onset, date of specimen collection, district name, province name, epidemiology number, test week, test month, test year, specimen type, referring laboratory name, test laboratory number, laboratory reference number, test registration date and test results review date.	Yes	
**Qualitative**			
Usefulness	Assessment of the system’s ability to meet its stipulated goals.		Yes
	Assessment of system’s ability to meet surveillance performance indicators as per WHO standards (calculation of annualised rubella and non-rubella febrile rash detection rate).	Yes	
Simplicity	Assessment of how key stakeholders feel about the rubella case definition, reporting process and tools of the system.		Yes
	Indirectly assessed by checking completeness of data collected through surveillance tools.	Yes	

CIFs: Case Investigation forms, IgM: Immunoglobulin M, WHO: World Health Organization

### Data collection

#### Quantitative

For retrospective rubella database analysis, we included all records of individuals who were tested for rubella infection from 2016 to 2018 in South Africa and excluded records that did not have test results.

#### Qualitative

We conducted face-to-face interview with individual participants using a structured paper-based questionnaire. The questionnaire had a mixture of close and open-ended questions and was comprised of two sections. First section included socio-demographic information and second one had questions based on each measured key attribute. Data collection took place over a two-week period. We explained the study and handed information sheet to the participants. Those who were interested to be part of the survey signed a written consent form before the interview.

### Data management and analysis

#### Quantitative

We received the rubella surveillance database from the CDW of the NHLS in a Microsoft Excel (2016) format. We analyzed the rubella surveillance data using Microsoft Excel and Stata (*Version 15*. StataCorp LLC, College Station, TX, United States of America, 2017).

To calculate the positive predictive value (PPV), we divided the total number of rubella IgM positive cases by the total number of suspect cases that were tested for rubella infection.

To assess system timeliness, we calculated facility and laboratory turnaround times and determined the proportion of samples that met the WHO desired turnaround times. WHO defines desired turnaround times as at least 80% of samples collected from the facilities arriving at the laboratory within three days, and at least 80% of the test results being disseminated to the national level from the laboratory within seven days of receipt of samples [23]. Specifically, for the facility turnaround time we divided the number of samples that were collected at the facilities and reached the laboratory within three days by the total number of rubella samples sent to the national laboratory by facilities. To determine the laboratory turnaround time, we divided the number of samples for which test results were disseminated to the national level within seven days after samples receipt by the total number of samples tested in the laboratory.

To determine the overall data quality of the system, we divided the total number of complete rubella records on the database by the total number of rubella records from 2016 to 2018. To determine the data quality of each variable on the database, we divided the total number of records with complete information for each variable by the total number of rubella records.

We assessed the system’s adequacy performance indicators by calculating rubella febrile rash detection rate for each year as the total number of rubella IgM positive cases divided by the South African mid-year population estimates of each year. We also calculated non-rubella febrile rash detection rate for each year as the total number of rubella negative cases divided by the South African mid-year population estimates. We obtained number of rubella cases from the rubella surveillance database and mid-year population estimates from statistics South Africa reports. We then compared the annual detection rates with the desired WHO rubella febrile rash and non-rubella febrile rash which is at least two cases per 100,000 population per year [23].

#### Qualitative

The attributes usefulness and simplicity were assessed based on participants responses to the relevant survey questions. We captured and processed survey response in Microsoft Excel. We compiled a summary report following information that we obtained from the participants.

#### Ethical considerations

The study was approved by the Faculty of Health Sciences Research Ethics Committee of the University of Pretoria (Ethic No 40/2019). We obtained approvals from Gauteng and Limpopo provincial DoH and management of all included health facilities. Rubella surveillance is part of the NICD’s routine surveillance activities and ethics approval for this was granted by the Human Ethics Research Committee (Medical) of the University of Witwatersrand (M210752). Survey participants read an information letter and signed a written consent form before participating in the study.

## Results

**Fig 1:**
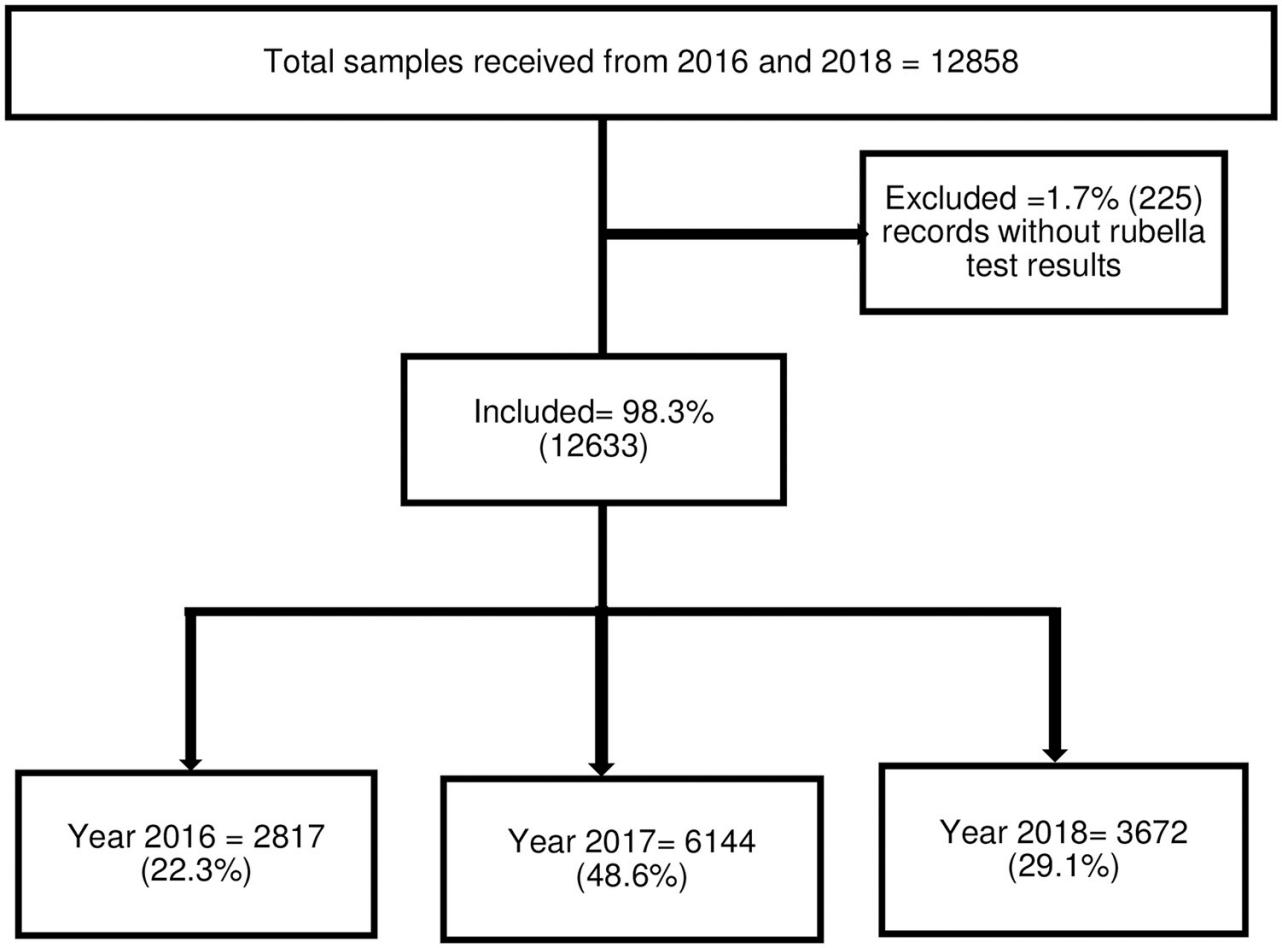
Number of samples tested for rubella infection in South Africa, 2016–2018.

A total of 12,858 samples were received and tested for rubella at the NICD laboratory from January 2016 to December 2018 (Fig 1). We excluded 1.7% (225/12,858) records that did not have rubella test results. Therefore, we included 98.3% (12,633/12858) of the records in our analysis. From the total 12,633 records, 48.6% (6144/12,633) were tested in 2017.

### Quantitative attributes

Positive predictive value: The overall PPV was 35.9% (4540/12633). The PPV was 29.1% (819/2816) in 2016, 40.9% (2512/6143) in 2017 and 32.9% (1209/3672) in 2018.

#### Timeliness

We described timeliness in terms of facility and laboratory turnaround times. The facility component had 70.5% (8903/12631) of timely reports, which is below the WHO target of 80% (Table 2). The laboratory component had 93.5% (11818/12631) timely report, almost 14% points above the WHO target.

**Table 2:** Facility and laboratory turnaround times of rubella surveillance system, South Africa, 2016–2018.

Turnaround times	Total N = 12631*	WHO recommended turnaround times*
**Facility**	**n (%)**	At least 80% of samples arriving at the laboratory within three days
Median days (range)	10 (0 to 163 days)	
≤3 days	8903 (70.5)	
4–163 days	3728 (29.4)	
**Laboratory**		At least 80% of the results disseminated to the national level from the laboratory within seven days
Median days (Range)	3 (0 to 202 days)	
≤7 days	11818 (93.5)	
8–202 days	813 (6.4)	

WHO: World Health Organisation.*WHO African regional guidelines for measles and rubella surveillance, revised April 2015 [23]. *Two records were excluded due to missing of sample taken and results review dates.

#### Data quality

The data management process starts at the health facilities continuing to the national level. Analysis of the rubella national database revealed that data quality of the system is poor, particularly information gathered at the facility-level. For the period under review, only 12.1% (1528/12633) of the records in the database were complete (Table 3). Data quality for information generated in the laboratory was good with more than 99% of complete records. District- and national-level participants also indicated that they receive incomplete surveillance tools, and they mostly receive these tools late from the facility-level. They further stated that incomplete records make it difficult for them to analyze data properly.

**Table 3:** Data quality of the rubella surveillance system, South Africa, 2016–2018.

Variables (N = 12633)	Records with information/N	Records without information/N
Overall data quality (2016–2018)	n (%)	n (%)
Complete records	1528 (12.1)	11105 (87.9)
**Data quality per year**		
2016	561 (4.4)	12072 (95.6)
2017	294 (2.3)	12339 (97.7)
2018	673 (5.3)	11960 (94.7)
**Clinical characteristics**		
Patient’s date of birth	12293 (97.3)	340 (2.7)
Sex	12353 (97.8)	280 (2.2)
Facility name	12632 (99.9)	1 (0.1)
Facility ward name	5089 (40.3)	7544 (59.7)
Date sample taken	12631 (99.9)	2 (0.1)
Date of symptoms onset	3752 (29.7)	8881 (70.3)
District name	12540 (99.3)	93 (0.7)
Epidemiology number	3277 (25.9)	9356 (74.1)
Province name	12631 (99.9)	2 (0.1)
**Laboratory characteristics**		
Test week	12633 (100)	0 (0.0)
Test month	12633 (100)	0 (0.0)
Test year	12633 (100)	0 (0.0)
Sample type	12624 (99.9)	9 (0.1)
Name of referring laboratory	12591 (99.7)	42 (0.3)
Test laboratory number	12631 (99.9)	2 (0.1)
Laboratory reference number	12633 (100)	0 (0.0)
Test registration date	12632 (99.9)	1 (0.1)
Results review date	12631 (99.8)	2 (0.2)

Rubella surveillance system adequacy performance indicators: Our findings showed that the system achieved the adequate surveillance indicator target since it was able to detect rubella and non-rubella febrile rash, apart from 2016 where the detection of rubella cases fell below the WHO-recommended targets (Table 4).

**Table 4:** Indicators of rubella surveillance system, South Africa, 2016–2018.

Surveillance indicators	Years under evaluation			
	2016	2017	2018	Target*
Annualised detection rate of rubella febrile rash illness	1.5	4.4	2.1	At least 2.0 per 100,000 population
Annualised detection rate of non-rubella febrile rash illness	3.6	6.4	4.3	At least 2.0 per 100,000 population

*WHO African regional guidelines for measles and rubella surveillance, revised April 2015 [23].

### Qualitative attributes

#### Usefulness

The analysis and interpretation of the surveillance data in the years under review and information gathered through the survey showed that the surveillance system was able to meet its stipulated goals (Table 5). Participants at the national level (NICD) indicated that they disseminate rubella surveillance data through a measles and rubella surveillance review publication once a year. They also stated that analysis of rubella surveillance data informs CRS sentinel surveillance. In addition, they indicated that the analysis of rubella surveillance data by age group showed that between 10 and 15% of cases occurred amongst females of reproductive age, which is useful in determining the at-risk population. Furthermore, participants mentioned that rubella immunoglobulin G (IgG) tests for residual samples is done to determine immunity gaps in different age groups within the South African population. However, participants at the facility level did not know of any decisions or policies that have been made or developed following rubella surveillance data.

**Table 5:** Objectives of rubella surveillance system, South Africa, 2016–2018.

Objectives*	Achieved
To estimate rubella incidence, prevalence, and trends over time	Yes
To detect rubella outbreaks	Yes
To identify populations at risk of rubella infection	Yes
To provide guidance to system users on decisions regarding rubella prevention and control strategies	Yes

*WHO African regional guidelines for measles and rubella surveillance, revised April 2015 [23].

#### Simplicity

Data for the rubella surveillance system is collected using tools such as notifiable medical conditions forms, case investigation forms, laboratory forms, and an electronic application. Surveillance information collected at the facilities is sent to the district, provincial, and national level daily using a combination of these tools. The national level does not send real time notification of rubella cases but sends weekly reports summarizing the surveillance data back to the provincial and district levels. Participants in both facility, district, and national levels were not certain if the system is simple. Our findings suggest that the system is not simple as there were several challenges raised regarding the operation of the system. Participants from the district and national level indicated that they receive incomplete information on cases from the facility level. The other aspect raised was that most of the rubella cases are asymptomatic, making it difficult for health care workers to identify all cases, and hindering the system from detecting all true cases. One participant said, “It is not simple to detect all rubella cases since we are currently dependent on measles case definitions for rubella surveillance”. Rubella surveillance is currently done concurrently with measles, and some cases could be missed as healthcare workers identify cases based on measles case definition”.

At the facility level, most of the participants did not know case definitions that are used and whether their facilities were conducting rubella surveillance. Participants also raised challenges regarding centralized laboratories and designation of personnel for sample collection. They said they are unable to send samples to laboratories over the weekend, and samples are not collected when designated personnel are off duty. Another issue raised was the confusion between the measles and rubella surveillance as these systems are operated concurrently. Participants from the district and facility level further raised challenges regarding data sharing. Participants indicated that they struggle to communicate with other stakeholders due to a poor or complete lack of internet connectivity at their facilities. As a result, they use their personal resources such as cellphones, data, and WhatsApp to communicate with other stakeholders. Participants at the national level (NICD) did not raise any challenges regarding sending rubella surveillance data to stakeholders and mentioned that the use of emails as means of communication is feasible and sustainable, since they are able to reach many people in a short period of the time.

## Discussion

We evaluated the rubella surveillance system in South Africa and found that the system was useful but not simple and had low PPV, poor timeliness and poor data quality at the health facility level. The surveillance system had a low PPV for the years under review. Since rubella is endemic in South Africa, we would expect to see a higher test positivity for cases reported to this surveillance system. The low PPV observed in our study could be because of the low sensitivity of the case definition that is used to identify cases in rubella surveillance in South Africa. This may imply that some rubella cases are missed given that samples tested for rubella are based on suspected measles case definition. This should be worrisome as the system may miss rubella outbreaks and underestimate incidence and prevalence.

The laboratory-component of the system showed commendable timeliness between the receipt of the samples in the laboratory and dissemination of results. The timeliness was higher than the WHO standard (80%) [23]. However, the systems’ facility component had timeliness below the WHO standard. Our findings are comparable to previous studies from Nigeria, Ghana, and Qatar where timeliness at the laboratory level surpassed WHO targets whereas at the health facility remained below the standard [26–28]. Poor timeliness at the facility-level may be explained by the fact that this surveillance system is laboratory-based and healthcare workers at health facilities are not well informed of it. Training involving both laboratory and health facility personnel, laying emphasis on awareness of the surveillance system could improve timeliness at facility level. Apart from the awareness of healthcare workers, it is possible that other challenges such as logistical issues might impact timeliness, though we did not assess this in our study. Poor timeliness at the facility-level could lead to delayed detection of outbreaks.

Analysis of the national rubella surveillance database showed poor data quality, particularly on the information that is gathered at the facility level. This is likely due to the facilities providing incomplete surveillance tools to both district, provincial and national levels. The provision of incomplete surveillance tools could be because healthcare workers at this level are not completely aware of the necessity of providing complete details of the suspect cases for surveillance system operation. Poor data quality at health facility level has been reported previously in South Africa [29, 30], and factors such as lack of training and knowledge of completing surveillance tools among healthcare workers have been cited as main contributors [31–33]. Although the database had incomplete records, data quality on the laboratory-component was commendable. This was expected since stakeholders in the laboratory component are well informed of this system compared to those at the facility-level. Enhanced training of healthcare workers on the notification process and roll-out of electronic medical records in health facilities could improve data quality at facility level. Contrary to our findings, good data quality at facility level was reported in Italy where they created mandatory variables on the notification form [34].

Regarding the usefulness of the rubella surveillance system, although stakeholders at national level (NICD) knew the importance of the system, those at district and facility levels did not know. Nonetheless, when comparing objectives of the system with the data provided in this study, and that of the national database, the system seems to be serving its purpose. For instance, the rubella national database can provide stakeholders with useful data to estimate the incidence and prevalence of rubella. The system also provides guidance to users for rubella prevention and control strategies. For example, the data collected by the rubella surveillance system were used to inform CRS sentinel surveillance in South Africa. As South Africa is preparing for the introduction of the rubella vaccine in the EPI, data from the rubella surveillance system may be useful in guiding authorities to prioritize vaccination of at-risk populations. The system met WHO-recommended surveillance indicators (≥ two cases detected per 100,000 population) [23] for the annual rubella rash detection rate in 2017 and 2018, and for non-rubella febrile rash detection rate from 2016 to 2018. While estimating rubella prevalence, incidence, and detecting rubella outbreaks are the primary objectives of this surveillance system, we expect superior attributes of PPV and timeliness. Low PPV and poor facility-level timeliness found in this study is of concern and may compromise the achievement of the systems’ objectives.

Our findings showed that stakeholders do not find the system simple to engage with. This is likely because rubella surveillance is solely laboratory-based and integrated into the measles surveillance system. Stakeholders are unable to differentiate the two systems, particularly those who are working at district- and facility-levels. Given the fact that confirmed rubella cases are only identified in the laboratory, there are no clear pathways for collecting and sharing data across the facility- and district-level. Most healthcare workers at the facility-level do not know case definitions that are used for this surveillance system. The facility-level timeliness that was below the WHO standard (80%) and a lack of knowledge about rubella surveillance case definitions support our conclusion that the system is not simple. Developing a clinical case definition for rubella would simplify the rubella surveillance system.

The system has several limitations that should be taken into consideration. The system is laboratory-based; therefore, healthcare workers at the facility-level do not know much about it. The system is conducted using measles case definitions; hence, some cases might be missed as healthcare workers focus on signs and symptoms of measles. Health facilities are not notified of the cases that test positive for rubella on real time and this might cause reluctance of healthcare workers to be involved in the system as they may not see the value of reporting suspected cases. Our study limitation was the inability to evaluate the system at WHO, provincial and national DoH levels as we were unable to reach key participants for interviews.

## Conclusions

We found that the system was useful based on the information gathered from the participants, analysis of rubella surveillance database and WHO indicators. However, the system was not simple, and had low PPV, poor timeliness and poor data quality at the facility level. Our findings highlighted the need to raise awareness among healthcare workers, particularly at the facility level to strengthen the functioning of this surveillance system. The use of rubella case definition and real-time notification of cases could improve PPV, timeliness and simplicity of the system. Future studies should assess factors that affect timeliness and data quality at the facility level. As South Africa considers introduction of the rubella vaccine in EPI schedule, it is essential that this surveillance system perform optimally.

## Supporting information

S1 File Manuscript data rubella database. (XLSX)

S2 File Manuscript data survey database. (XLSX)
